# The relationship between empathy and mental health: A mediation model via rumination and interpersonal competence

**DOI:** 10.3389/fpsyt.2025.1725496

**Published:** 2025-12-17

**Authors:** Fan Zhang, Chenqi Jiang, Tszching Wong, Yachang Lin, Ching-yi Ho, Yen-wen Chen, Meiyan Poon, Siuhin Li, Xueting Wang, Yuting Fang, Xinhua Li, Pengjun Li, Yuying Ma

**Affiliations:** 1 Department of Public Health and Preventive Medicine, School of Medicine, Jinan University, Guangzhou, Guangdong, China; 2 The Graduate School, Jinan University, Guangzhou, Guangdong, China; 3 Jinan University Organization Department, Jinan University, Guangzhou, Guangdong, China; 4 Health Science Center of Jinan University, Jinan University, Guangzhou, China

**Keywords:** empathy, medical students, psychological distress, rumination, interpersonal competence

## Abstract

**Background:**

As a critical capacity in medical education, the trajectory of empathy development and its relationship with medical students’ mental health has remained inconclusive. To address this purpose, the current study was conducted, with addressing the mediation role of rumination and interpersonal competence in this relationship.

**Methods:**

A total of 640 medical students from Guangdong Province, China, were recruited. The levels of empathy across grades and genders were assessed. In addition, participants’ interpersonal competence, rumination, and psychological distress were measured. Linear regression assessed the empathy–distress association, and parallel mediation modeling tested rumination and interpersonal competence as simultaneous mediators.

**Results:**

In total, 523 participants were included in the final analysis (with 44% were male). Group comparisons showed that male students reported higher empathy than females (84.69 ± 9.84 vs. 82.68 ± 8.58, *p* = 0.016); and higher-grade students performed higher empathy (*p* < 0.001). Empathy was positively associated with psychological distress, more rumination thinking, and better interpersonal competence. Interpersonal competence (*β* = -0.01, *p* = 0.018) and rumination (*β* = 0.02, *p* = 0.016) significantly fully mediated the relationship between empathy and distress. Empathy was associated with increased rumination and better interpersonal competence; however, the first linked to higher distress and the latter was associated with reduced distress.

**Conclusion:**

Empathy in medical students associates with greater psychological distress through dual pathways: heightened rumination (intrapersonal) and impaired interpersonal competence (interpersonal). The findings have highlighted the need for tailor-made empathy training programs, differentiated by gender and grade, that integrate coping strategies and interpersonal skills development.

## 1 Introduction

As a multi-faceted construct, empathy was defined as the social, cognitive, and affective processes involving the capacity to understand other’s emotions or reflecting other’s concerns (1). Benefits of empathy were found in various domains for healthcare professionals. For example, medical students with higher empathy showed better mental health and better communication skills (2). Empathy was also found to benefit social adaptation and clinical practice for doctors (3). Far from being merely a “soft skill”, empathy is a therapeutic necessity for high-quality patient-centered care. Therefore, cultivating empathy in medical students has become a priority in medical education. However, despite the widely-documented importance of empathy, how it affects medical students’ mental health has remained inconclusive, which may create barriers in developing educational strategy.

Increased attention has been paid to the empathy development during medical undergraduate education, while the findings has remained mixed. Hojat et al. (4, 5) first reported the decline in empathy among medical students. However, Colliver et al. (2010) has found inconsistent results which triggered extensive debate (6, 7). Studies in France (8) and in Span (9) reported stable or even increased empathy, suggesting potential cultural or curricular influences. Evidence from longitudinal studies were also inconsistent. Archer & Turner (10) found that students’ perceived empathy has increased across 4 years during the undergraduate study in medical school; while with a two-year follow-up, Bhatia & Shetty (11) reported significant decline of MBBS students’ empathy level. Even among Chinese medical students, how empathy develops in medical school has been inconclusive. Wen et al. (12), and Ye et al. (13) reported an upward trend in empathy levels, whereas Li et al. (14); Wang et al. (15) found opposite results, with a decline in empathy identified across academic years. A multicenter cross-sectional study revealed that the university category was a significant factors that affects medical students’ empathy (16). In addition, the variations in curricular design and academic pressure across institutions, could also contribute to the heterogeneity in the findings. Up to today, no conclusion could be reached regarding the development of empathy across undergraduate years in medical training (17–19), while emerging evidence suggested that multiple factors such as curriculum settings, prioritization of biomedical knowledge, student’s prior personal values and professional experiences were related with the changes in empathy (20, 21). Another inconclusive finding in medical students’ empathy lies in gender difference. While a large number of studies demonstrated female reported greater competence of understanding others (9, 22–24), some research found no gender difference (14, 25), or even opposite results such that male showed higher level of empathy (16).

**Figure 1:**
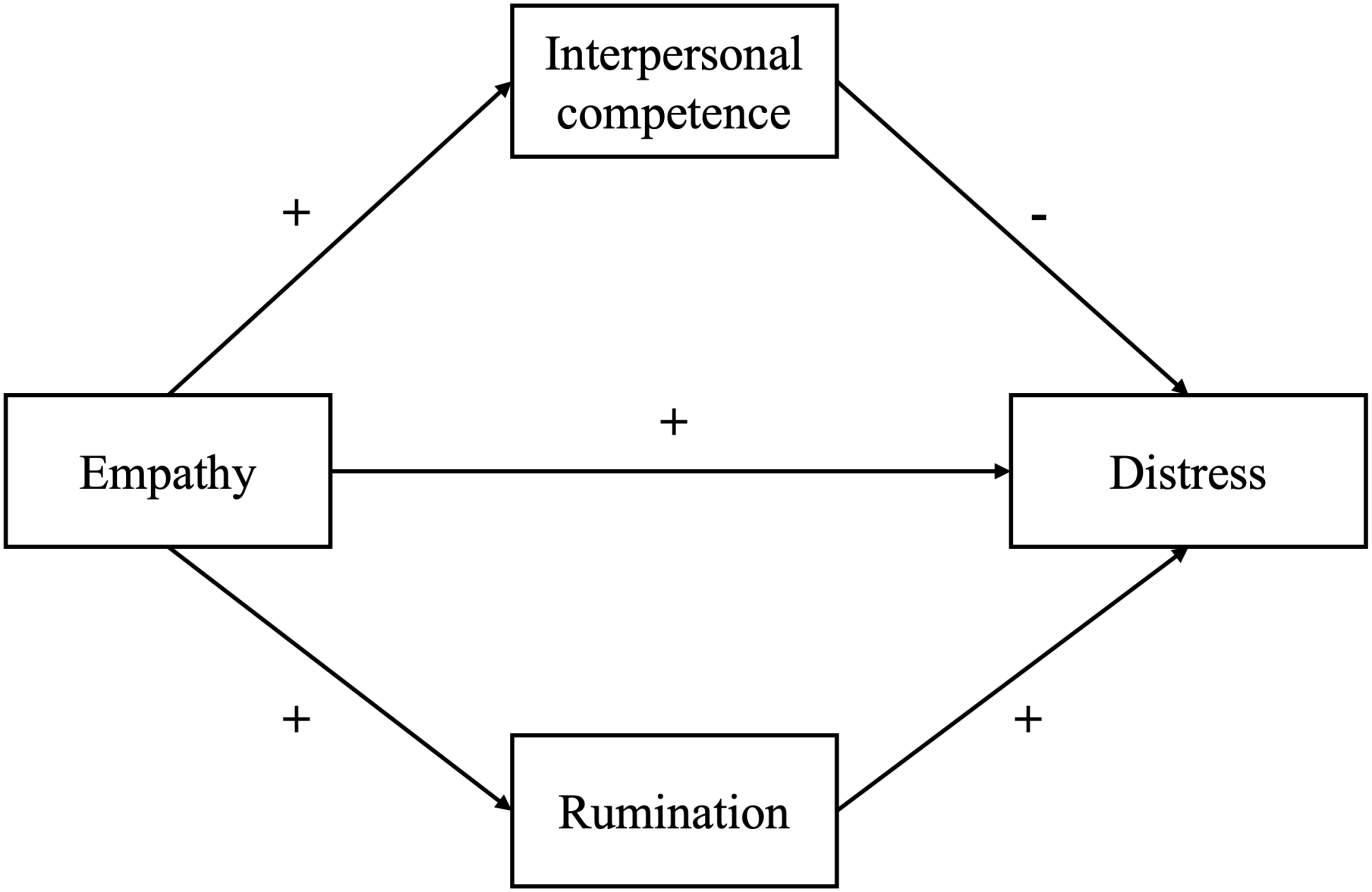
Hypothesized model of parallel mediation model.

Empathy not only affects the relationship with patients and professional performance, but also affects medical student’s mental health. Existing literature has found that empathy may have a double-edged sword effect on medical student’s or physician’s mental health. In particular, cognitive empathy was negatively related with depressive symptoms, while affective empathy showed positive associations (26, 27). Individuals with higher level of cognitive empathy are more likely to attend to other’s negative emotions (28, 29), which can lead to psychological distress through rumination (29). Rumination, defined as a pattern of repetitive and prolonged negative thinking about the self and one’s distress (30), plays a key role in the effect of empathy on mental health. According to the Response Styles Theory, rumination was a key mechanism in the development and maintenance of depression (31). Meanwhile, affective empathy was found to be is associated with better interpersonal competence, which indicate better ability to perceive and manage social interactions (32). This competence typically manifests as strengthened social connectedness and increased social support (33), both were recognized as crucial protective factors for mental health (34). For medical students, although those with high empathy could better perceive patient’s suffering and experience greater distress, at the same time, better interpersonal competence may help establish better patient-doctor relationship and reduce conflicts (35). Therefore, it is necessary to take both intrapersonal pathway (e.g., rumination) and interpersonal pathway (e.g., interpersonal competence) into consideration when understanding the effects of empathy on medical student’s mental health (see **Figure 1**).

The current study has a two-fold purpose: first, to address the changes of empathy among medical students across sexes; second, to address the effects of empathy on medical student’s mental health with including rumination and interpersonal competence as mediators. According to the previous literature, it is hypothesized that 1) empathy may decrease during undergraduate education, and female students’ empathy is usually higher than male students; 2) the direction of how empathy is related with mental health has been unclear, because greater empathy is associated with greater interpersonal competence, it could also link to higher rumination, which cast contradictory effects on mental health. Therefore, a mediation model including rumination and interpersonal competence was developed to address the relationship between empathy and mental health. The findings would provide insights for building empathy training programs in undergrade medical education, promoting the career development and mental health for medical students.

## 2 Methods

### 2.1 Participants

A total of 640 medical students participated in this study. Participants were mainly recruited from Jinan University in Guangdong, China. As a university with over 50% non-local students (i.e., from Hong Kong/Macao, Taiwan, and other regions and countries), the students pool can better represent a mixed sample of students from mainland and other Chinese regions. Inclusion criteria were: (1) Undergraduate and graduate students majoring in clinical medicine, stomatology, and traditional Chinese medicine; (2) No history of mental illness. Those who were on psychotropic medications were excluded. Furthermore, to ensure data quality, the responses were excluded if it (1) failure to correctly answer the attention-check question, which serves to identify inattentive respondents and enhance the validity of self-report data (36); or (2) completion of the survey in less than 100 seconds, as such a short response time may indicate careless or random responses (37). After the exclusion, a total of 523 responses (81.72%) were included in the analysis. All the participants provided informed consent before taking part in the survey.

### 2.2 Measure

The participants’ demographic characteristics (i.e., age, sex, grade, major, place of origin) were asked. In addition, the following factors were assessed with well-established measures.

*Empathy*. The Chinese version of Jefferson Empathy Scale for medical students (JSE-S) (36) was used to assess students’ empathy. JSE-S has been widely adopted in medical research to measure self-reported empathy among medical students. This instrument employs a 7-point Likert scale (1=strongly disagree to 7=strongly agree), with total scores ranging from 20 to 140; higher scores indicate greater empathy. Half of items (2, 3, 7, 9, 12, 14, 15, 17, 18, 20) were reverse items, which were reversely coded when scoring. The Cronbach’s alpha was 0.75, indicating good reliability.

*Rumination*. The 13-item Chinese version of Ruminative Responses Scale (RRS-CV) (37, 38) was adopted, which consists of domains: symptom rumination, reflection and deep thinking, and compulsive meditation,. Participants were asked to rate using a Likert-type scale, ranging from 1 (strongly disagree) to 7 (strongly agree). The higher total score indicate a higher tendency of rumination. The Cronbach’s alpha of the overall scale is 0.90.

*Interpersonal competence*. The 15-item Chinese Version of the Brief Interpersonal Competence Questionnaire (ICQ-15 (31, 39), was adopted. It has 5 dimensions: initiation of a relationship, negative assertion, disclosure, emotional support, and conflict management. The participants rated each item according to how challenging it was for them, with a 5-point Likert scale from 1 “I am extremely poor at this” to 5 “I am extremely good at this”. Higher scores indicate better interpersonal competence. The Cronbach’s alpha of the overall scale was 0.93.

*Mental health.* Patient Health Questionnaire-2 (PHQ-2) was adopted to assess depressive symptoms and Generalized Anxiety Disorder 2-item (GAD-2) for anxiety symptoms (40). Each served as a preliminary screening for depression or anxiety, with higher scores suggesting more severe risk for the specific mental disorder. Previous studies have found good reliability and validity for both measurements. In the current study, The Cronbach’s alpha of PHQ-9 was 0.80, and the Cronbach’s alpha of GAD-2 was 0.87. To indicate overall level of psychological distress, the scores of PHQ-2 and GAD-2 were combined.

### 2.3 Statistical analysis

The minimum number of participants required was determined by an *a priori* power analysis (Gpower: (41)). According to GPower 3.1, to detect a small interaction effect equivalent to *f*^2^ = 0.05 at the final step with a power of 0.80 and an alpha of 0.05, a sample size of 402 individuals would be sufficient. Considering a 20% missing rate, at least 503 participants should be recruited. The data analysis included t-test, analysis of variance (ANOVA), chi-square tests, and linear regression analysis, which was performed using R 4.3.2. The R-package mediation version 4.5.0 was used for mediation analysis.

## 3 Results

### 3.1 Descriptive results of the sample

In total, 523 participants were included in the data analysis, which fulfilled the sample size requirement. There were 293 females (56.02%) and 230 males (43.98%). Considering the curriculum schedule, students were categorized into three grade level: 20.08% were in lower grade (year 1, before taking systematic anatomy course), 52.96% in middle grade (year 2–3, before hospital internship), and 26.96% were in senior years (Grades 4–6 and graduate school). As shown in **Table 1**, male showed significant higher level of empathy compared with female (male: 84.69 ± 9.84, female: 82.68 ± 8.58; *p* = 0.016). Regarding the differences across grades, lower grade showed lower level of empathy compared with middle or senior grade participants (lower grade: 80.75 ± 6.37, middle grade: 84.25 ± 9.25, senior grade: 84.21 ± 10.43, *p* < 0.001). Another significant group difference was found in rumination, with lower grade showed higher level of rumination compared with middle or senior grades (lower grade: 72.07 ± 11.60, middle grade: 67.19 ± 12.39, senior grade: 64.52 ± 13.09, *p* < 0.001).

**Table 1:** The descriptive results of empathy, interpersonal competence, rumination and distress.

Variables (n)	Empathy	*p-value*	Interpersonal competence	*p-value*	Rumination	*p-value*	Distress	*p-value*
Sex								
Female (293)	82.68 ± 8.58	0.02	49.64 ± 9.83	0.73	68.07 ± 12.12	0.203	3.60 ± 3.00	0.87
Male (230)	84.69 ± 9.84		49.34 ± 9.20		66.63 ± 12.80		3.65 ± 2.98	
Grade								
Lower (105)	80.75 ± 6.37	<0.001	50.33 ± 9.05	<0.001	72.07 ± 11.60	< 0.001	3.47 ± 2.79	0.81
Middle (277)	84.25 ± 9.25		49.08 ± 10.27		67.19 ± 12.39		3.63 ± 2.92	
Senior (141)	84.21 ± 10.43		49.74 ± 8.45		64.52 ± 13.09		3.73 ± 3.27	
Major								
Clinical Medicine (413)	83.60 ± 9.33	0.63	49.50 ± 9.63	0.89	67.73 ± 12.66	0.695	3.75 ± 3.05	0.29
Oral Medicine (80)	83.72 ± 8.75		49.31 ± 9.87		66.77 ± 11.69		3.37 ± 2.78	
Traditional Chinese Medicine (13)	84.17 ± 11.54		50.25 ± 8.63		65.58 ± 11.47		2.75 ± 2.77	
Preventive Medicine (12)	80.91 ± 3.65		49.00 ± 7.94		67.73 ± 11.41		2.64 ± 2.11	
Others (5)	77.75 ± 4.35		54.25 ± 3.40		59.75 ± 7.81		1.75 ± 2.87	
Place of origin								
Mainland (233)	82.79 ± 8.50	0.11	49.44 ± 9.56	0.88	67.34 ± 12.95	0.837	3.38 ± 2.83	0.11
HK/MAC/TW (290)	84.11 ± 9.59		49.57 ± 9.58		67.57 ± 12.00		3.82 ± 3.10	

HK, Hongkong; MAC, Macao; TW, Taiwan.

To further understand the group differences in empathy, the interaction effect between sex and grade were tested. It was found that the interaction was significant (*F* = 3.41, *p* < 0.05). Simple effect analysis revealed that among male students, senior grade showed significantly higher level of empathy compared with that of lower grade (87.46 vs. 81.17, *p* < 0.001); while among female, middle grade showed higher level of empathy than that of lower grade (83.92 vs. 80.39, *p* = 0.041).

Linear regression model was run to test the effect of empathy on rumination, interpersonal competence, and distress. It was found that empathy was positively associated with more distress (*β* = 0.03, *p* = 0.049). Meanwhile, empathy was also associated with higher level of rumination (*β* = 0.20, *p* < 0.001) and better interpersonal competence (*β* = 0.20, *p* < 0.001), see **Table 2**. Moreover, rumination and interpersonal competence was regressed to predict participant’s psychological distress. It was found that only rumination was associated with more distress (*β* = 0.08, *p* < 0.001), while the relationship between interpersonal competence and distress was not significant. For details, see **Table 3**.

**Table 2:** Total effect of empathy on interpersonal competence, rumination, and distress.

Variables	Interpersonal competence			Rumination			Distress		
	*β* ± sd	Std. *β* ± sd	*p-value*	*β* ± sd	Std. *β* ± sd	*p-value*	*β* ± sd	Std. *β* ± sd	*p-value*
(Intercept)	34.24 ± 3.91	0.18 ± 0.11	< 0.001	0.49 ± 0.11	56.55 ± 5.00	< 0.001	-0.02 ± 0.11	1.09 ± 1.24	0.381
Empathy	0.20 ± 0.05	0.20 ± 0.05	< 0.001	0.15 ± 0.04	0.20 ± 0.06	< 0.001	-0.09 ± 0.05	0.03 ± 0.02	0.049
Grade (lower)									
middle	-2.01 ± 1.12	-0.21 ± 0.12	0.075	-0.46 ± 0.12	-5.71 ± 1.44	< 0.001	-0.02 ± 0.12	0.06 ± 0.36	0.875
senior	-1.33 ± 1.26	-0.14 ± 0.13	0.292	-0.67 ± 0.13	-8.33 ± 1.61	< 0.001	-0.05 ± 0.13	0.16 ± 0.4	0.699
Sex (female)									
male	-0.79 ± 0.87	-0.08 ± 0.09	0.366	-0.16 ± 0.09	-2.04 ± 1.11	0.067	-0.004 ± 0.09	-0.01 ± 0.28	0.966

**Table 3:** Total effects of interpersonal competence/rumination on distress.

Variables	Distress			Variables	Distress		
	*β* ± sd	Std. *β* ± sd	*p-value*		*β* ± sd	Std. *β* ± sd	*p-value*
(Intercept)	3.78 ± 0.79	-0.06 ± 0.11	0.6	(Intercept)	-2.37 ± 0.83	-0.20 ± 0.10	0.054
Interpersonal competence	-0.01 ± 0.01	-0.02 ± 0.05	0.639	Rumination	0.08 ± 0.01	0.33 ± 0.04	< 0.001
Grade (lower)				Grade (lower)			
middle	0.16 ± 0.35	0.05 ± 0.12	0.662	middle	0.56 ± 0.34	0.18 ± 0.11	0.099
senior	0.26 ± 0.4	0.09 ± 0.13	0.522	senior	0.87 ± 0.39	0.29 ± 0.13	0.025
Sex (female)				Sex (female)			
male	0.05 ± 0.28	0.02 ± 0.09	0.859	male	0.18 ± 0.26	0.06 ± 0.09	0.492

### 3.2 The mediation model of empathy

**Figure 2:**
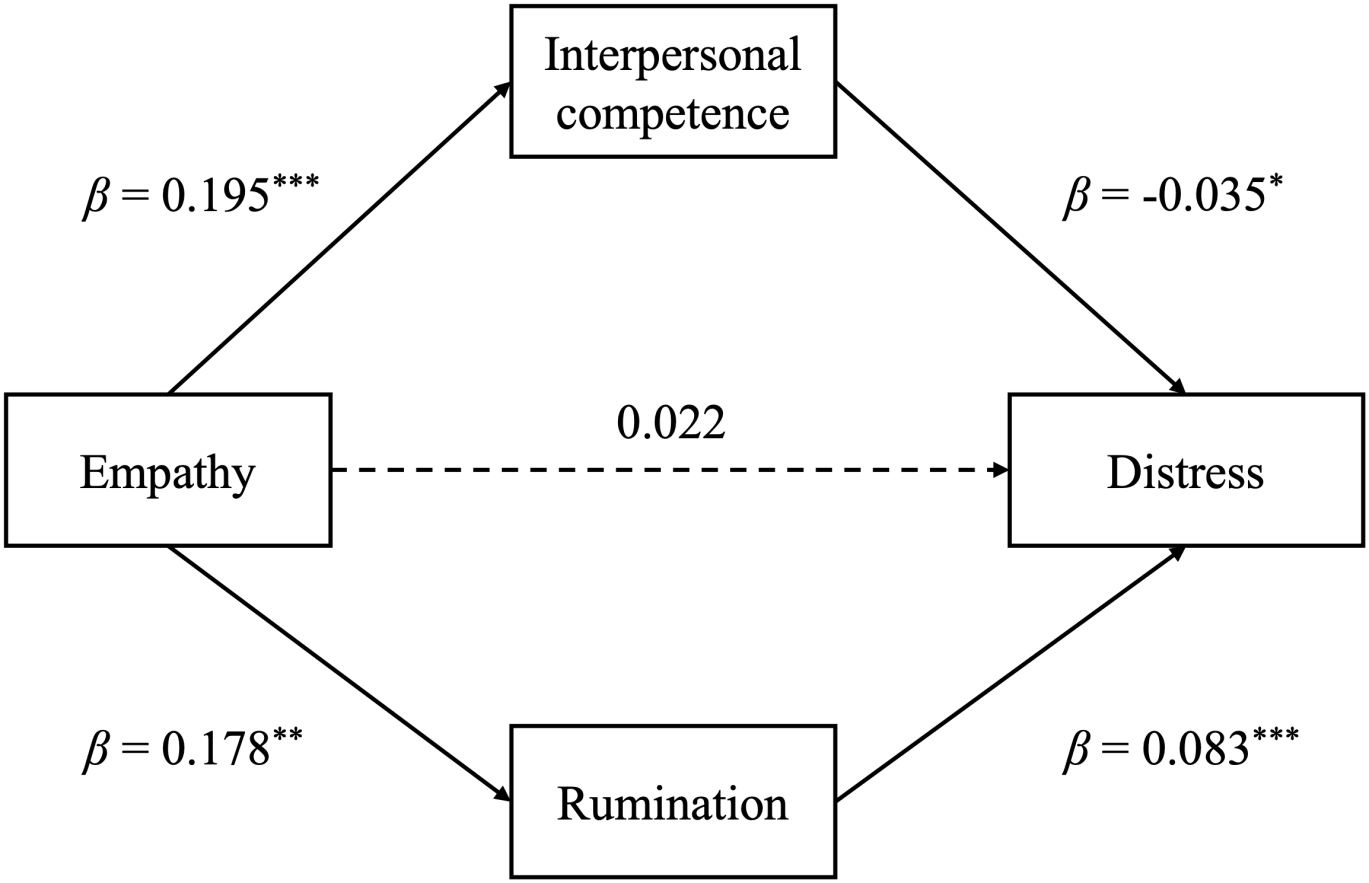
Parallel mediation model of empathy, interpersonal competence, rumination and distress. Sex and grade were set as control variables for the model. Statistical significance levels are indicated as follows: *p <.05, **p <.01, ***p <.001.

According to the results of linear regression, the total effect of empathy on psychological distress is significant (*β* = 0.03, *p* = 0.049). The parallel mediation model was run with empathy as the independent variable (x), interpersonal competence/rumination as the mediator (M), and psychological distress as the dependent variable (y). Sex and grade were entered in the models as covariate. 5000 bootstrap samples were used. As shown in **Figure 2**, after including interpersonal competence and rumination, the direct effect of empathy on distress was not significant (*β* = 0.02, *p* = 0.132). Meanwhile, empathy was positively associated with interpersonal competence (*β* = 0.20, *p* < 0.001), which was further related to less distress (*β* = -0.04, *p* = 0.013). Thus, the impact of empathy on distress was mediated by interpersonal competence (*β* = -0.01, *p* = 0.018). In contrast, empathy was positively associated with rumination (*β* = 0.18, *p* = 0.003), which was related to more distress (*β* = 0.08, *p* < 0.001). It suggested that rumination has also mediated the effect of empathy on distress (*β* = 0.02, *p* = 0.016), though the direction of effect on psychological distress was opposite to interpersonal competence.

## 4 Discussion

With a sample of 523 medical students, how empathy changes across sex and grade were tested and how empathy affects mental health and the potential mediation pathways were addressed. The results showed that perceived empathy has generally increased with grade, with male showing higher level of empathy. Meanwhile, empathy was associated with heightened distress, which were fully mediated by increased rumination thinking and interpersonal competence. Parallel mediation showed that rumination and interpersonal competence both mediated the effect of empathy on distress, though the direction of effect were opposite.

The results showed that middle and high grades students reported higher levels of empathy than lower grade students. Though the results can hardly solve the questions whether empathy increases or decreases across years in medical school, it has provided nuanced evidence that the trajectory of empathy may not be linear in medical school, and the curriculum setting may play a critical role in empathy development. In consistent, recent study of Archer & Turner (10) has found that across three time points assessing empathy, students’ empathy increased at the second follow-up (year 4), while fell to the same level at the last follow-up (year 6). Samarasekera et al. (42) has found similar results, such that empathy was the lowest in year 5, and highest in year 2. Our results also suggested that the significant increase in empathy may occur in year 2, though the level has remained till senior years. It is possible that different curriculum across regions may lead to different patterns in empathy, pinpointing that there might be a critical time window to deliver empathy interventions. Regarding why empathy may decline in medical school, researchers have proposed two key factors: the increased complexity of clinical cases and the influence of the ‘hidden curriculum’, characterized by excessive workload pressures, an overemphasis on biomedical knowledge, and exposure to poor clinical role models (43). Therefore, more attention should be paid to the students who started internship or residency, to help them cope with the complex clinical situation and remain empathetic towards the patients. It is also possible that differences in curriculum structure between schools and countries may contribute to the inconsistent findings in empathy development (53), which reminds us that the programs to train student’s empathy should be tailor-made with considering the specific situation of each university.

Gender difference in empathy was also found in the current study, such that male showed higher empathy than female. The findings were different from prior research that typically reported higher levels of empathy in females (8, 44, 45). This discrepancy could be contributed to social, cultural and educational factors. It was suggested that social factors, such as changes in gender role expectations, are the primary driver of observed differences (46, 47). As Lopez-Zafra & Garcia-Retamero indicated (48), evolved social norms and the popularity of gender equality ideologies may explain the changes in empathy expression across genders. Recent studies have found no significant difference in empathy across genders (13, 14, 49). On the other hand, cultural factors may also influence the gender difference in empathy. For example, research in Asia, such as China (14), Iran (50) and Pakistan (51), found no significant gender differences in empathy, suggesting that higher level of empathy among female may be more pronounced in western culture. Therefore, when understanding gender difference in empathy, it is necessary to consider factors such as cultural concepts, social norms, and religious beliefs. In line with our findings, a recent multi-center survey in China also found that female medical students had lower levels of empathy (16). The authors suggested it is possible that this may reflect a tendency of female to experience greater personal distress when empathizing, prompting them to downregulate empathic engagement. In addition, both Huang et al. (2024) (16) (mean = 67.38) and the present study (mean = 83.52) reported relatively low overall empathy level compared with prior research (mean ranged from 104 to 109) (12, 52), and whether this is due to methodological factors or represents a recent trend has remained unclear.

This study has several limitations to be acknowledged. First, the cross-sectional design prevents us from drawing causal inferences or examining the developmental trajectory of empathy across medical training. Longitudinal research with repeated, preferably annual, assessments is needed to more accurately capture within-student changes over time and to clarify the causal mechanisms underlying empathy development. In addition, longitudinal evidence would also help address the non-linear trajectory of empathy, and identify the most optimal and potentially sensitive periods for implementing targeted empathy-enhancement training. Second, because the study was conducted in Guangdong, the extent to which the findings can be generalized to medical students into other regions of China or internationally remains uncertain. Future studies should employ larger, more geographically diverse samples to improve external validity. Finally, qualitative or mixed-methods approaches, such as in-depth interviews or focus group discussions, could be employed to further explore the reasons behind the higher empathy levels observed in male students.

## 5 Conclusion

In conclusion, this study found that empathy increased with academic grade and was higher in male medical students. In addition, empathy exhibited a dual effect on mental health: on the one hand, it is associated with more psychological distress via increased rumination thinking; one the other hand, it is related with reduced distresses via increased interpersonal competence. Our findings highlighted the double-edged sword effect of empathy in medical training. Therefore, education and interventions should not only cultivate empathy but also pay more attention to developing coping strategies and interpersonal skills, to safeguard students’ mental well-being and promote professional development.

## Data Availability

The raw data supporting the conclusions of this article will be made available by the authors, without undue reservation.
